# Epidemiology of injuries among children and adolescents from the Xinglin District in Xiamen, 2016–2019

**DOI:** 10.3389/fped.2024.1387761

**Published:** 2024-09-02

**Authors:** Gangxi Lin, Qiyi Zeng

**Affiliations:** ^1^ Zhujiang Hospital, Southern Medical University/The Second School of Clinical Medicine, Southern Medical University, Guangzhou, Guangdong, China; ^2^ Department of Pediatrics, School of Medicine, The First Affiliated Hospital of Xiamen University, Xiamen, China

**Keywords:** children, adolescents, injuries, epidemiology, fall-related injuries, mechanical injuries, China

## Abstract

**Objective:**

To investigate the epidemiology of injuries among children and adolescents in the Xinglin District of Xiamen from 2016 to 2019.

**Methods:**

This study collected data from patients who attended the outpatient and emergency departments of the Xinglin District at the First Affiliated Hospital of Xiamen University between January 2016 and December 2019, diagnosed with injuries.

**Results:**

A total of 13,123 patients were included, categorized into age groups 0–4 (*n* = 4,834), 5–9 (*n* = 3,924), 10–14 (*n* = 2,671), and 15–18 (*n* = 1,694). The rates of unintentional injuries were 97.00%, 96.94%, 94.50%, and 90.08% in the 0–4, 5–9, 10–14, and 15–18 age groups, respectively (*P* < 0.001). The proportion of head injuries decreased with age (from 41.13% in the 0–4 age group to 18.00% in the 15–18 age group), compensated by an increase in rates of injuries to upper and lower extremities and multisite injuries (*P* < 0.001). The most common causes of injuries were fall-related injuries (30.46%–52.05%), followed by mechanical injuries (18.35%–36.42%), with the rates of fall-related injuries decreasing with age and rates of mechanical injuries increasing with age (*P* < 0.001). Age-period-cohort models revealed that the time factor was not significant for fall-related injuries and mechanical injuries (all *P* > 0.05) despite apparent increases in incidence over time.

**Conclusion:**

Injuries in children and adolescents continue to be a significant public health concern in the Xinglin District (China), predominantly driven by fall-related injuries and mechanical injuries.

## Background

The impact of injuries exacts an excessive toll on both the economy and human lives, as evidenced by the substantial direct and indirect costs amounting to $29.4 billion in Canada in 2018 (1). In China, the financial burden is similarly alarming, with estimated annual direct costs of injury reaching US$10 billion (2), accompanied by a staggering annual loss of 12.6 million years of productivity (3). Children and adolescents emerge as an especially susceptible population to the detrimental effects of injuries due to their small stature and ongoing physical development (4). This vulnerability manifests in the alarming statistic that injuries rank as a leading cause of both morbidity and mortality in this age group (5). Shockingly, injuries not only claim the unfortunate distinction of being the primary cause of death among children aged 1–15 years in China but also the fourth leading cause of death in infants under one year (6). Ensuring an equitable outcome for all injured individuals in this demographic is not just a matter of societal concern; it has become a critical public health issue that demands immediate attention and comprehensive intervention (7).

The leading causes of injury in children and adolescents encompass fall-related injuries, sports accidents, and vehicle injuries (8). Although sports-related injuries are prevalent in this demographic, access to sports is often associated with a higher socioeconomic status, thereby influencing the epidemiology of sports injuries (8). The economic development in China over the past few decades has notably elevated the socioeconomic status of many families, reshaping how children and adolescents engage in sports activities. Concurrently, China has experienced a rapid transformation in transportation trends over the last decade, marked by a significant increase in privately owned cars. This shift has altered the epidemiology of vehicle accidents and related injuries (9, 10), leading to a 4.7% increase in car crashes and a 3.1% rise in vehicle injury deaths for every 10% increment in gross domestic product (11). A comprehensive study examining the epidemiology of injuries in China from 1990 to 2017, irrespective of patient age, disclosed noteworthy trends. Despite a decline in disability-adjusted life years (DALYs) and mortality rates, there was a concurrent increase in the incidence of injuries (10).

Determining the burden of injuries in children and adolescents in China holds significant importance as a public health imperative, crucial for both strategic planning and contextualizing economic trends and health interventions. Accurate data on injuries are essential to inform the development of policies, regulations, and laws for injury prevention and control in China (12). However, there is a notable gap in recent data specifically focused on children and adolescents. Particularly noteworthy is the impact of the previous one-child policy, which resulted in heightened parental attention and an inadvertent overprotection of children. This shift contributed to notable improvements in injury epidemiology within the young age groups (12). Yet, the extent to which the subsequent changes to the two-child (13) and three-child (14) policies have influenced injury epidemiology in children remains unknown.

Therefore, this study aimed to examine the epidemiology of injuries among children and adolescents from the Xinglin District in Xiamen from 2016 to 2019.

## Materials and methods

### Study design and patients

This study collected data from patients who attended the outpatient and emergency departments of Xinglin District (a rural-urban continuum) of the First Affiliated Hospital of Xiamen University between January 2016 and December 2019. The study was approved by the Ethics Committee of the First Affiliated Hospital of Xiamen University, which waived the requirement for informed consent due to the nature of the retrospective study. The hospital is the only tertiary general hospital in the study area, serving approximately 400,000 residents. It has 500 beds and receives 600,000 patients annually, including 120,000 children. It is almost always the first choice for injured patients in the study area.

The inclusion criteria were (1) patients aged <19 years old and (2) with confirmed cases of injuries according to the International Classification of Diseases Version 10 (ICD10) (update and revision 2019) (Supplementary Material S1). The exclusion criteria were: (1) The patients combined with iatrogenic injuries, (2) duplicate records within 3 months, and (3) patients with incomplete data. The patients were divided into four age groups: 0–4 years old (including those aged <5 years old), 5–9 years old (those age ≥5 years old and age <10 years old), 10–14 years old (those age ≥10 years old and age <15 years old), and 15–18 years old (those age ≥15 years old and age <19 years old) (15).

### Data collection

Data for the study were obtained from patients' medical records, including outpatient and emergency medical records from the hospital's electronic medical record system and medical report cards from the National Injury Surveillance System (NISS) in China. The data were from a single hospital, but the NISS data for that single hospital were used in this study. Therefore, all data used in this study underwent the quality control process of NISS. Upon admission of an injured patient, the physicians and administrative staff complete the case report form, which is submitted, reviewed, and validated by the NISS before entry into the database.

Four main data elements were collected: (1) demographic characteristics (sex and age), (2) basic characteristics of the injury (admission year, admission season, injury causes, injury location, activities at the time of injuries, and whether the injury was intentional), (3) clinical characteristics on the injury (injuries nature, injuries body parts and systems, injuries severity, clinical diagnosis, and the outcomes of the injuries), and (4) injury-involved item characteristics (name of the items causing injuries, and relationship of the injuries to the items’ use).

The severity of the injuries was classified as mild, moderate, and severe. Mild injuries are relatively low in severity, do not interfere greatly with physical functions and daily activities, and generally do not require hospitalization or surgical intervention. Such injuries may include minor abrasions, contusions, or sprains. Moderate injuries are moderate in severity and, to a certain extent, impact physical functions and daily activities and may require some medical intervention and rehabilitation therapy. Such injuries may include fractures, cuts, or moderate contusions. Severe injuries are those that are excessively severe, resulting in a substantial impairment of physical functions and daily activities, and generally require immediate medical assistance and a protracted course of rehabilitation therapy. Such injuries may involve severe fractures, deep cuts, or serious internal injuries by penetrating or non-penetrating trauma.

The injuries were disposed of based on the two clinical classification conventions, as shown in Supplementary Materials S2.

### Statistical analysis

All statistical analyses were conducted using R 4.1.2 software. Continuous variables were presented as means ± standard deviations (SD). Categorical variables were expressed as *n* (%) and analyzed using the chi-squared test. The subgroup analysis was conducted among children with the two most prevalent major categories of injuries [fall-related injuries and mechanical injuries (injuries due to external physical forces)]. Differences were considered statistically significant at a two-sided *P* < 0.05.

Epidemiological cohort studies, retrospective or prospective observational analyses of morbidity or mortality rates for a given disease, are done to explore the effects of cohort factors on morbidity or mortality rates. For this reason, Kermark and Mckendrick (16) proposed the cohort effect model to study the change in mortality rate. Later, Frost (17) presented the age-period-cohort (APC) model to analyze the influence of the age effect and period effect on the change in mortality or morbidity. This model is based on the Poisson model, simultaneously adjusting the effects of age, period, and cohort on the outcome, and addresses the shortcomings of the traditional descriptive analysis that can only calculate the occurrence rate of a certain time and its time change trend but cannot eliminate the effects of the interactions among age, period, and cohort. In the APC model, the age effect refers to the risk of an event due to the age of the individual patients. The period effect refers to the risk variation caused by the age groups in different periods or years, which includes a series of social, cultural, economic, and natural environment changes to the outcome. The cohort effect refers to the different impacts on individuals or groups due to different ages of experiencing various types of events, i.e., at various stages of life due to the experience of different social or historical events that make each birth cohort of populations have different exposure risks to social, economy, behavioral, and environmental factors (18).

The basic expression form of the model is:

Therein, *R_ijk_* indicates the prevalence of a type of unintentional injury attributable to children with the *j* period in the *i* age group in the *k*^th^ birth cohort; *u* indicates the intercept of the regression equation; *a_i_* indicates the age effect parameter; *b_j_* indicates the period effect parameter, and *y_k_* indicates the cohort effect parameter.

This study used the “APC” package from the R 4.1.2 software to conduct exploratory comparative analyses of annual percent changes for the section and through the Akaike information criterion (AIC), −2 log-likelihood, and its chi-squared test results to evaluate the fitting degree. The effect coefficient in the model was calculated to reflect the relative risk (RR). An effect coefficient <0 indicates a decrease in risk, and an effect coefficient >0 is an increase in risk. The larger the value, the higher the risk. The joinpoint regression analysis involves fitting a series of joined straight lines on a log scale to the trends in the annual age-adjusted incidence. Line segments are joined at points called joinpoints. Each joinpoint denotes a statistically significant change in trend. Stratification analysis was conducted to establish APC models for fall-related injuries and mechanical injuries, stratified by sex.

### Patient and public involvement

No patient involved.

## Results

### Characteristics of the patients

**Figure 1:**
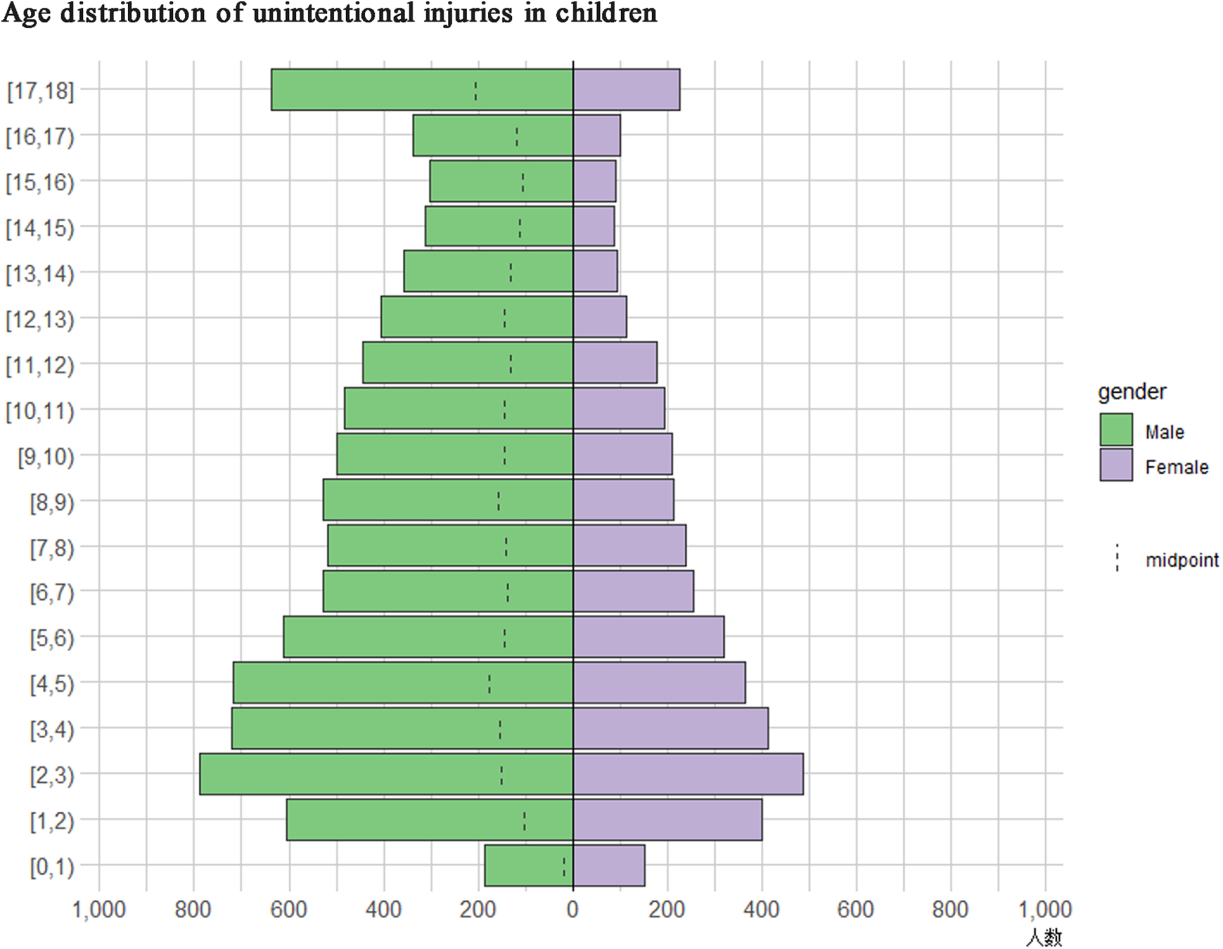
Age pyramid graph of injuries among children and adolescents. *Y*-axis: age distribution of unintentional injuries among children and adolescents. *X*-axis: the number of children and adolescents. The distribution of the injuries are shown as *n* (%).

After excluding duplicate records (*n* = 1,208), patients with iatrogenic injury (*n* = 4), and patients with incomplete data (*n* = 103), a total of 13,123 patients were included. These patients were divided into the following age groups: 0–4 (*n* = 4,834), 5–9 (*n* = 3,924), 10–14 (*n* = 2,671), and 15–18 (*n* = 1,694). There were 8,974 males and 4,149 females. The percentage of males increased with age, from 62.39% in the 0–4 age group to 75.27% in the 15–18 age group (*P* < 0.001). A total of 3,599, 3,329, 2,330, and 3,865 patients were included from 2016, 2017, 2018, and 2019, respectively. The number of injuries was significantly lower in 2018 (*P* < 0.001). A total of 3,878, 3,355, 3,470, and 2,420 injuries occurred in spring, summer, fall, and winter, respectively, with the smallest number being in winter (*P* < 0.001) (Table 1). Figure 1 presents the distribution of the injuries for males and females for each year of age.

**Table 1:** Baseline characteristics of the injuries among children and adolescents.

	0–4 years old	5–9 years old	10–14 years old	15–18 years old	*P*
	*n* = 4,834	*n* = 3,924	*n* = 2,671	*n* = 1,694	
Gender					<0.001
Male	3,016 (62.39%)	2,682 (68.35%)	2,001 (74.92%)	1,275 (75.27%)	
Female	1,818 (37.61%)	1,242 (31.65%)	670 (25.08%)	419 (24.73%)	
Age, years	2.34 ± 1.23	6.88 ± 1.43	11.73 ± 1.4	16.54 ± 1.11	–
Consultation time					
Year					<0.001
2016	1,476 (30.53%)	1,038 (26.45%)	608 (22.76%)	477 (28.16%)	
2017	1,244 (25.73%)	1,004 (25.59%)	631 (23.62%)	450 (26.56%)	
2018	819 (16.94%)	717 (18.27%)	506 (18.94%)	288 (17.00%)	
2019	1,295 (26.79%)	1,165 (29.69%)	926 (34.67%)	479 (28.28%)	
Season					<0.001
Spring (March–May)	1,403 (29.02%)	821 (30.74%)	526 (31.05%)	1,128 (28.75%)	
Summer (June–August)	1,333 (27.58%)	546 (20.44%)	417 (24.62%)	1,059 (26.99%)	
Fall (September–November)	1,181 (24.43%)	813 (30.44%)	444 (26.21%)	1,032 (26.3%)	
Winter (December–February)	917 (18.97%)	491 (18.38%)	307 (18.12%)	705 (17.97%)	

Continuous variables were presented as means ± standard deviations (SD). Categorical variables were expressed as *n* (%) and analyzed using the chi-squared test.

### Characteristics of the injuries

The proportions of unintentional injuries were 97.00%, 96.94%, 94.50%, and 90.08% in the 0–4, 5–9, 10–14, and 15–18 age groups, indicating an increase in intentional injuries with age (*P* < 0.001). The proportion of head injuries decreased with age (from 41.13% in the 0–4 age group to 18.00% in the 15–18 age group), compensated by an increase in the rates of injuries to upper and lower extremities and multisite injuries (*P* < 0.001). Most injuries involved the motor system, with increasing rates with age (from 39.37% in the 0–4 age group to 60.63% in the 15–18 age group), compensated by a decrease in central nervous injury with age (from 22.49% in the 0–4 age group to 10.74% in the 15–18 age group) (*P* < 0.001). Most injuries were contusions/abrasions (31.05%–36.55%). Fractures were the highest in the 10–14 age group (18.20%) and the lowest in the 0–4 age group (7.20%), while sprains/strains were the highest in the 10–14 age group (16.02%) and the lowest in the 5–9 age group (11.01%) (*P* < 0.001). Injury severity and the rate of hospitalization increased with age (both *P* < 0.001) (Supplementary Table S1).

The most common causes of injuries were fall-related injuries (30.46%–52.05%), followed by mechanical injuries (18.35%–36.42%), with the rates of fall-related injuries decreasing with age and the rates of mechanical injuries increasing with age (*P* < 0.001). Most injuries occurred during leisure activities (22.96%–55.83%). In most cases, the injuries occurred when normally performing the activity or using the product when the accident occurred. In the 0–4 and 5–9 age groups, most injuries occurred at home (69.34% and 36.67%), while most injuries in the 10–14 and 15–18 age groups occurred at school (34.14% and 20.48%) (*P* < 0.001) (Supplementary Table S2).

### Subgroup analysis

The characteristics of the patients with fall-related injuries are shown in Supplementary. Among patients with fall-related injuries, there were 2,516 (40.46%), 1,847 (29.70%), 1,339 (21.53%), and 516 (8.30%) patients in the 0–4, 5–9, 10–14, and 15–18 age groups, respectively. The proportion of males increased with age (*P* < 0.001). The proportion of injuries at home decreased with age, while the proportion of injuries at school or during sports increased (*P* < 0.001). The proportion of injuries during leisure activities decreased with age, while the proportion of injuries during physical activity increased (*P* < 0.001). The severity of the injuries increased with age (*P* < 0.001). The proportion of injuries involving the head decreased with age, while those affecting the limbs increased (*P* < 0.001). Accordingly, the proportion of injuries affecting the motor system increased with age, while the injuries affecting the central nervous system decreased (*P* < 0.001). Contusions and abrasions were the main nature of injury in the 0–4 age group, while sprains/strains were the major nature in the 15–18 age group (*P* < 0.001) (Supplementary Table S3). The effects varied across different ages, periods, and cohorts, with some fluctuations over time. The risk of children falling and getting injured decreased gradually from ages 0 to 9, but there was a slight increase at age 10, followed by a decline until age 14. However, the highest effect was observed at age 0 (0.264), followed by ages 14 (0.189) and 12 (0.186).

Regarding the period effects, the highest effect was observed in 2019 (0.145). Looking at cohort effects from birth, there was not a consistent pattern in the risk of children falling and getting injured. Nevertheless, overall, children born before 2007 generally had lower risk effects compared with those born after. The highest risk effects were observed in children born in 2010 (0.132), followed by those born in 2013 (0.082) and 2014 (0.074) (Table 2).

**Table 2:** Analysis results of the morbidity of the fall-related injuries in the age-period-cohort model.

	Coefficient	Std. err.	*z*	*P* > |*z*|	95%CI
Age (years)					
age_0	0.264	0.071	3.700	<0.001	(0.124, 0.404)
age_1	0.179	0.042	4.270	<0.001	(0.097, 0.261)
age_2	0.089	0.035	2.570	0.010	(0.021, 0.157)
age_3	0.084	0.031	2.680	0.007	(0.022, 0.145)
age_4	0.084	0.030	2.840	0.005	(0.026, 0.143)
age_5	0.079	0.032	2.460	0.014	(0.016, 0.142)
age_6	−0.024	0.038	−0.620	0.532	(−0.099, 0.051)
age_7	−0.042	0.043	−0.980	0.326	(−0.126, 0.042)
age_8	−0.108	0.048	−2.230	0.026	(−0.203, −0.013)
age_9	−0.017	0.051	−0.330	0.740	(−0.117, 0.083)
age_10	0.039	0.054	0.720	0.472	(−0.067, 0.145)
age_11	0.108	0.058	1.870	0.061	(−0.005, 0.221)
age_12	0.186	0.063	2.970	0.003	(0.063, 0.309)
age_13	0.083	0.070	1.190	0.234	(−0.054, 0.221)
age_14	0.189	0.071	2.650	0.008	(0.049, 0.328)
age_15	−0.092	0.081	−1.140	0.253	(−0.25, 0.066)
age_16	−0.247	0.090	−2.750	0.006	(−0.422, −0.071)
age_17	−0.290	0.107	−2.720	0.007	(−0.499, −0.081)
age_18	−0.566	0.131	−4.320	<0.001	(−0.822, −0.309)
Period (years)					
period_2016	−0.064	0.017	−3.860	<0.001	(−0.096, −0.031)
period_2017	−0.084	0.015	−5.710	<0.001	(−0.112, −0.055)
period_2018	0.002	0.018	0.140	0.891	(−0.032, 0.037)
period_2019	0.145	0.015	9.530	<0.001	(0.115, 0.175)
Cohort (years)					
cohort_1998	−0.167	0.244	−0.680	0.493	(−0.646, 0.311)
cohort_1999	−0.197	0.156	−1.260	0.207	(−0.503, 0.109)
cohort_2000	−0.194	0.139	−1.390	0.164	(−0.466, 0.079)
cohort_2001	0.015	0.092	0.160	0.874	(−0.165, 0.195)
cohort_2002	−0.049	0.089	−0.540	0.587	(−0.224, 0.126)
cohort_2003	−0.033	0.085	−0.390	0.699	(−0.199, 0.134)
cohort_2004	0.001	0.080	0.010	0.990	(−0.156, 0.158)
cohort_2005	−0.103	0.075	−1.370	0.172	(−0.251, 0.045)
cohort_2006	0.020	0.067	0.300	0.764	(−0.111, 0.152)
cohort_2007	−0.021	0.065	−0.320	0.748	(−0.147, 0.106)
cohort_2008	0.039	0.059	0.660	0.509	(−0.076, 0.153)
cohort_2009	0.057	0.054	1.060	0.291	(−0.049, 0.164)
cohort_2010	0.132	0.051	2.610	0.009	(0.033, 0.232)
cohort_2011	0.056	0.045	1.240	0.213	(−0.032, 0.145)
cohort_2012	0.047	0.037	1.260	0.209	(−0.026, 0.12)
cohort_2013	0.082	0.030	2.700	0.007	(0.022, 0.141)
cohort_2014	0.074	0.027	2.800	0.005	(0.022, 0.126)
cohort_2015	0.043	0.026	1.620	0.105	(−0.009, 0.094)
cohort_2016	0.039	0.032	1.210	0.226	(−0.024, 0.101)
cohort_2017	0.013	0.039	0.330	0.745	(−0.064, 0.09)
cohort_2018	0.010	0.053	0.190	0.846	(−0.093, 0.114)
cohort_2019	0.135	0.148	0.910	0.363	(−0.156, 0.426)
cons	−0.821	0.020	−42.030	<0.001	(−0.859, −0.782)

The age-period-cohort (APC) model analyzes the influence of the age effect and period effect on the change in mortality or morbidity. The age effect refers to the risk of an event due to the age of the individual patients. The period effect refers to the risk variation caused by the age groups in different periods or years. The cohort effect refers to the different impacts on individuals or groups due to different ages of experiencing various types of events.

**Figure 2:**
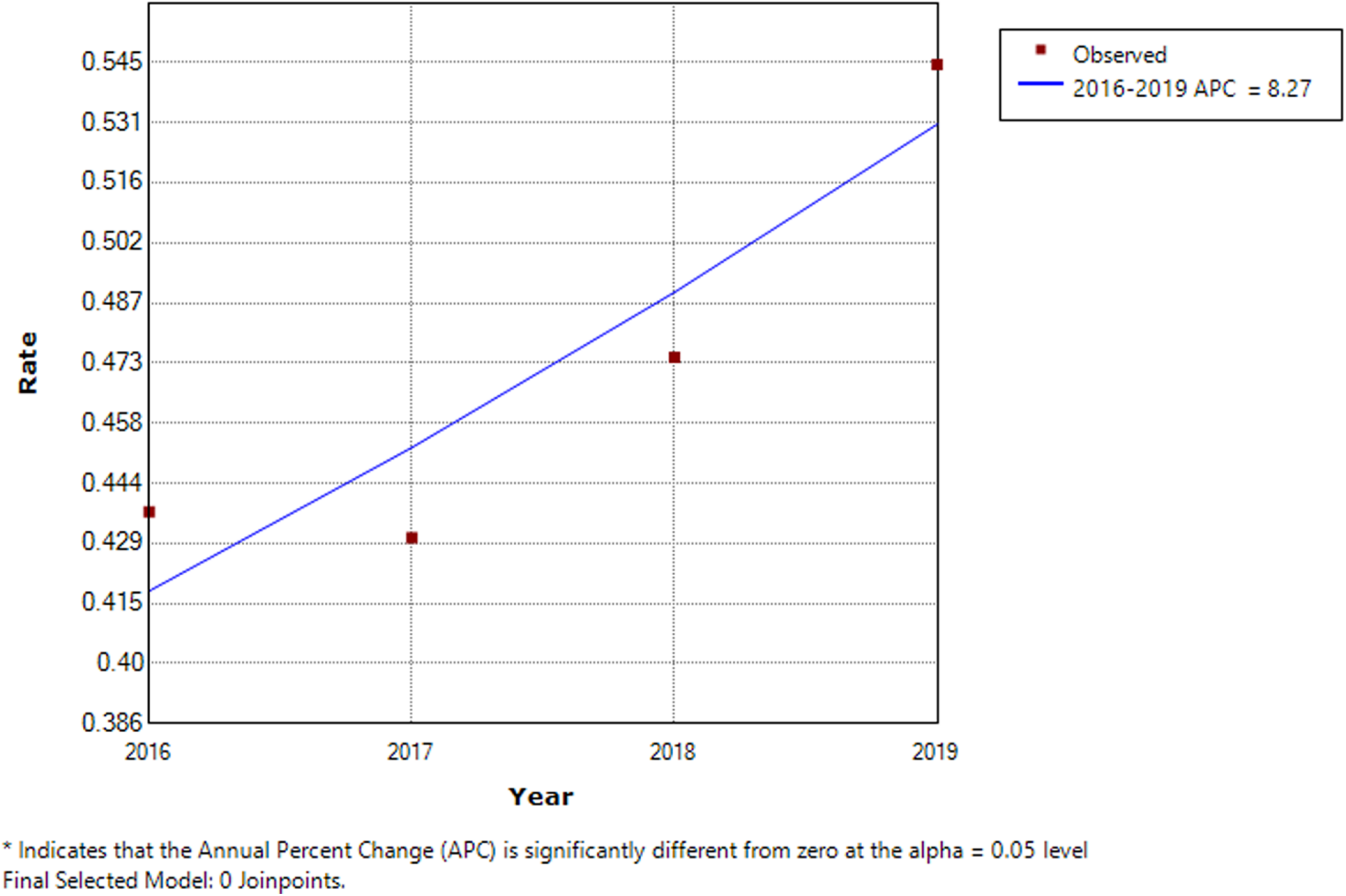
Trend analysis results of the joinpoint regression model applied to fall-related injuries. The joinpoint regression analysis involves fitting a series of joined straight lines on a log scale to the trends in the annual age-adjusted incidence. Line segments are joined at points called joinpoints. Each joinpoint denotes a statistically significant change in trend.

Figure 2 shows that the prevalence rate showed a gentle upward trend throughout the study period. The figure shows that there is no turning point (i.e., the number of joinpoints was 0), meaning that the prevalence trend of the four years has not changed, and all showed an upward trend. The annual percent changes of the prevalence of mechanical injuries in children from 2016 to 2019 was 8.27%. Because there was no turning point, the annual percent changes during the whole study period from 2016 to 2019 was also 8.27%, i.e., the prevalence rate of fall-related injuries in children increased at an average rate of 8.27% per year from 2016 to 2019 (all *P* > 0.05). Although the P-value was >0.05, the increasing rate of incidence could be used for reference. The lack of statistical significance may be related to the short period. The stratification analysis showed similar results, with annual percent changes of fall-related injuries at 8.43% (with no joinpoints, *P* > 0.05) in males and 7.91% (with no joinpoints, *P* > 0.05) in females.

Among patients with mechanical injury (Supplementary Table S4), the proportion of males increased with age (*P* < 0.001). The proportion of intentional injuries increased with age (*P* < 0.001). The proportion of injuries at home decreased with age, while the proportion of injuries at school, during sports, or in industry/construction regions increased (*P* < 0.001). The proportion of injuries during leisure activities decreased with age, while the proportion of injuries during physical activity and at work increased (*P* < 0.001). The severity of the injuries increased with age (*P* = 0.002). The proportion of injuries involving the head decreased with age, while those affecting the upper limbs increased (*P* < 0.001). Accordingly, the proportion of injuries affecting the motor system increased with age, while the injuries affecting the central nervous system decreased (*P* < 0.001). Most injuries were from sharp instruments, bite injuries, or open injuries in all age groups, increasing with age; contusions and abrasions decreased with age (*P* < 0.001).

Similar to the findings in fall-related injuries, the effects varied across different ages, periods, and cohorts, with some fluctuations over time. The risk of children getting injured due to external physical forces showed a fluctuating trend in age effects, rising from ages 0 to 9, showing a slight decline at age 10, followed by an increase until age 14, and then peaking at age 17 (0.367), followed by ages 18 (0.361) and 16 (0.311).

Regarding the period effects, the effects were consistently positive from 2017 to 2019, indicating an increase in risk, with the highest effect being observed in 2018 (0.070). Looking at the cohort effects from birth, there was not a consistent pattern in the risk of children falling and getting injured. The highest risk effects were observed in children born in 1998 (0.419), followed by those born in 1999 (0.360) and 2002 (0.214). Nevertheless, for cohorts born after 2006, there was a general decrease in the risk effects of mechanical injury (Table 3).

**Table 3:** Analysis results of the morbidity of the mechanical injuries in the Age-period-cohort model.

	Coefficient	Std. err.	*z*	*P* > |*z*|	95%CI
Age					
age_0	−0.492	0.239	−2.060	0.039	(−0.96, −0.024)
age_1	−0.084	0.088	−0.950	0.342	(−0.257, 0.089)
age_2	−0.220	0.075	−2.920	0.004	(−0.367, −0.072)
age_3	−0.193	0.073	−2.640	0.008	(−0.335, −0.05)
age_4	−0.107	0.068	−1.580	0.114	(−0.24, 0.026)
age_5	−0.094	0.078	−1.200	0.229	(−0.247, 0.059)
age_6	0.036	0.085	0.430	0.668	(−0.13, 0.203)
age_7	0.136	0.088	1.540	0.124	(−0.037, 0.309)
age_8	0.207	0.095	2.170	0.030	(0.02, 0.394)
age_9	0.206	0.105	1.970	0.049	(0.001, 0.411)
age_10	−0.015	0.117	−0.130	0.896	(−0.245, 0.214)
age_11	−0.037	0.122	−0.300	0.764	(−0.277, 0.203)
age_12	−0.197	0.142	−1.390	0.165	(−0.474, 0.081)
age_13	−0.082	0.142	−0.580	0.564	(−0.359, 0.196)
age_14	−0.215	0.151	−1.430	0.154	(−0.51, 0.08)
age_15	0.088	0.126	0.700	0.482	(−0.158, 0.335)
age_16	0.331	0.110	3.020	0.002	(0.117, 0.546)
age_17	0.367	0.104	3.530	0.000	(0.163, 0.571)
age_18	0.361	0.106	3.400	0.001	(0.153, 0.57)
Period (years)					
period_2016	−0.155	0.036	−4.350	0.000	(−0.225, −0.085)
period_2017	0.019	0.029	0.660	0.510	(−0.038, 0.076)
period_2018	0.070	0.036	1.930	0.053	(−0.001, 0.142)
period_2019	0.065	0.031	2.100	0.036	(0.004, 0.127)
Cohort (years)					
cohort_1998	0.419	0.144	2.900	0.004	(0.136, 0.702)
cohort_1999	0.360	0.114	3.170	0.002	(0.138, 0.583)
cohort_2000	0.188	0.117	1.600	0.109	(−0.042, 0.418)
cohort_2001	0.145	0.108	1.340	0.179	(−0.067, 0.358)
cohort_2002	0.214	0.118	1.810	0.070	(−0.017, 0.446)
cohort_2003	−0.122	0.148	−0.820	0.410	(−0.413, 0.169)
cohort_2004	0.011	0.154	0.070	0.942	(−0.291, 0.314)
cohort_2005	0.061	0.154	0.390	0.694	(−0.242, 0.363)
cohort_2006	−0.088	0.146	−0.600	0.547	(−0.373, 0.198)
cohort_2007	−0.063	0.141	−0.450	0.653	(−0.339, 0.212)
cohort_2008	−0.138	0.128	−1.080	0.281	(−0.388, 0.113)
cohort_2009	−0.057	0.119	−0.480	0.633	(−0.29, 0.176)
cohort_2010	−0.204	0.120	−1.700	0.089	(−0.438, 0.031)
cohort_2011	−0.099	0.107	−0.920	0.357	(−0.309, 0.111)
cohort_2012	−0.072	0.097	−0.740	0.460	(−0.263, 0.119)
cohort_2013	−0.062	0.091	−0.680	0.498	(−0.24, 0.116)
cohort_2014	−0.074	0.088	−0.840	0.398	(−0.245, 0.098)
cohort_2015	−0.001	0.084	−0.020	0.988	(−0.165, 0.163)
cohort_2016	−0.027	0.096	−0.280	0.782	(−0.215, 0.162)
cohort_2017	0.062	0.106	0.580	0.563	(−0.147, 0.27)
cohort_2018	0.079	0.134	0.590	0.555	(−0.183, 0.341)
cohort_2019	−0.533	1.107	−0.480	0.630	(−2.703, 1.636)
cons	−1.491	0.057	−26.140	0.000	(−1.603, −1.379)

The age-period-cohort (APC) model analyzes the influence of the age effect and period effect on the change in mortality or morbidity. The age effect refers to the risk of an event due to the age of the individual patients. The period effect refers to the risk variation caused by the age groups in different periods or years. The cohort effect refers to the different impacts on individuals or groups due to different ages of experiencing various types of events.

**Figure 3:**
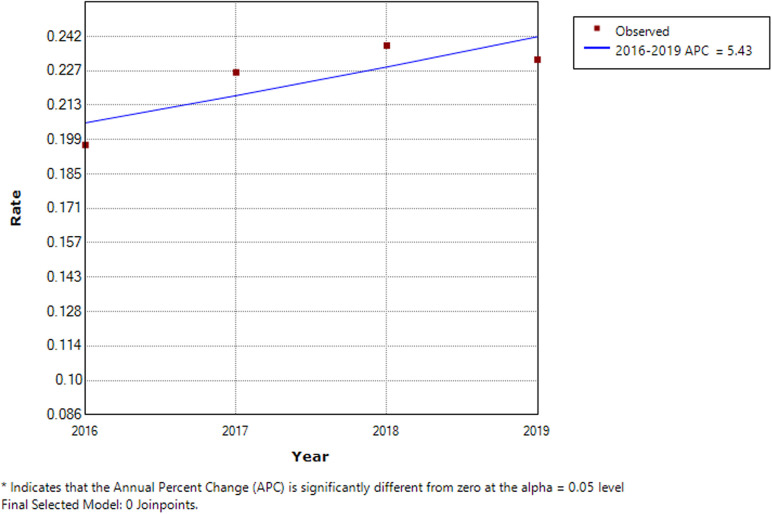
Trend analysis results of the joinpoint regression model applied to mechanical injuries. The joinpoint regression analysis involves fitting a series of joined straight lines on a log scale to the trends in the annual age-adjusted incidence. Line segments are joined at points called joinpoints. Each joinpoint denotes a statistically significant change in trend.

In the same way as for Figure 2, Figure 3 shows that the prevalence rate showed a gentle upward trend throughout the study period. There is no turning point (i.e., the number of joinpoints was 0), meaning that the prevalence trend of the four years has not changed, showing an upward trend. The annual percent changes of the prevalence of mechanical injuries in children from 2016 to 2019 was 5.43%. Because there was no turning point, the annual percent changes during the whole study period from 2016 to 2019 was also 5.43%, i.e., the prevalence rate of mechanical injury in children increased at an average rate of 5.43% per year from 2016 to 2019 (all *P* > 0.05). Although the *P*-value was >0.05, the increasing rate of incidence could be used for reference. The lack of statistical significance may be related to the short period. The stratification analysis showed similar results, with annual percent changes of mechanical injuries at 4.11% (with no joinpoints, *P* > 0.05) in males and 8.88% (with no joinpoints, *P* > 0.05) in females.

## Discussion

This study underscores the persistent significance of injuries in this population, highlighting the pressing nature of the public health challenge within the Xinglin District, China. The predominant contributors to injury incidence were fall-related injuries and mechanical injuries, although while the proportion of these two categories increased over the years, the observed trends did not reach statistical significance. Indeed, the effects varied across different ages, periods, and cohorts, and although the overall effect was not significant, the differences revealed the complexity of the factors affecting children's injuries. These results offer valuable insights that can inform policy formulation aimed at mitigating the occurrence of injuries in Xiamen, with potential applicability to regions facing similar conditions.

The prevalence of injury in children and adolescents has been reported as 17.3% in Europe (19), while a higher prevalence of injury was observed in Southeast Asian countries at 42.2% (20). Although the prevalence was not determined in the present study, a meta-analysis of 187 studies in China reported a pooled prevalence of 23% of injuries among children and adolescents (21). Still, comparison among studies is difficult due to differences in injury definition, data collection methods, and cultural and lifestyle characteristics (20). Still, the most important factor for the difference in the prevalence of injury is the socioeconomic status of the countries, with a high injury burden observed in low- and moderate-income countries, which often display weak infrastructures for safety and regulations and poor societal response to injuries (22, 23). A previous meta-analysis of Chinese data (published up to 2017) suggested that child injury showed a decreasing trend in recent years (21), supporting the decrease observed in 2018 in the present study. This decrease could be related to the implementation of surveillance and prevention programs in the 2010s in China, as observed in previous studies (24–27). Indeed, three of those studies included the implementation period of the programs and showed changes in the epidemiology of injuries (24–26), while the fourth study showed the patterns of injury from before program implementation (27).

When observing the incidence of injuries by age and sex, it could be noted that most injuries occurred in males and in the 0–4 age group. The predominance of males for injuries has been observed in several studies (28–30). Boys are more likely to be involved in dangerous activities than girls, and girls may be checked more closely by their guardians than boys. Hence, efforts should be taken to decrease the rates of injuries in boys. A previous meta-analysis showed that the rates of injuries increased with age, while the present study showed that most injuries occurred in the 0–4 age group, as supported by a study in China that reported a high rate of injuries in children 0–6 years (31). Children 0–4 years of age are more susceptible to fall-related injuries (the most frequent injury in the present study) because of unstable gait and poor coordination. In addition, the guardians might be more susceptible to consult for injuries in infants and young children.

In the present study, fall-related injuries were the most common type of injury, followed by mechanical injuries (i.e., those due to inanimate objects or plants), and both types of injury. It is supported by a meta-analysis of Chinese studies and other Chinese studies published after the meta-analysis was conducted (21, 32), as well as by community-based studies in various countries (33, 34). fall-related injuries are responsible for the loss of >6.8 million DALYs worldwide (35). The Global Burden of Disease indicates that 172 million fall-related injuries yearly result in short- or long-term disability worldwide (36). Importantly, many fall-related injuries are preventable, and a study in Canada showed that taking effective prevention measures can decrease the occurrence of fall-related injuries by 20% among children <10 years old (35). Considering the importance of fall-related injuries as a cause of injury in children and adolescents, proper measures against fall-related injuries should be taken in China. Still, a trend toward an increase in the incidence of fall-related injuries could be noted over the study period (i.e., from 2016 to 2019). The present study was not designed to determine the reasons for such increases, especially in the era of injury prevention. The two- and three-child policies could play a role, i.e., the parents have to divide their attention among their children, increasing the risk of fall-related injuries. Still, the exact causes should be investigated, and if possible, preventive actions and policies should be implemented.

Mechanical injuries include all injuries due to objects, devices, machines, etc. The present study showed that the rates of mechanical injuries increased with age, and age was statistically significant in the APC model. It is probably related to the children starting to use toys and various tools for cooking, playing in the garden, etc. In addition, the results showed that the proportions of mechanical injuries at work and injuries in industry/construction regions increased with age, consistent with the fact that several children eventually started to work to help their families. It is supported by a study in China that showed that the rate of mechanical injury increased with age (37). Interestingly, the same study in China showed that the rate of mechanical injury in children decreased from 2002 to 2010, probably related to better safety measures and the safe design of tools, devices, and machines over time. Still, the present study observed a trend toward an increase in the incidence of mechanical injuries from 2016 to 2019. As for fall-related injuries, the present study could not determine the causes of that increase. Again, divided attention among children could be involved. Changes in socioeconomic factors could also be involved. According to numbers from the United Nations, 218 million children worldwide were working in 2020, including 73 million in hazardous work or conditions (38), and the problem is particularly apparent in poorer countries (39). The International Labor Organization reports 10 million injured children at work each year, with 22,000 deaths (40). Nevertheless, the results strongly suggest that children's safety when working with tools, devices, and machines should be improved and enforced.

Of note is that the present study showed that the rate of intentional injury increased with age, reaching about one in 10 injuries in the 15–18 age group and that the greatest part of that increase was due to the increase in intentional mechanical injuries. A multi-country study showed that adolescents aged 14–17 had rates of intentional injuries of 8.90% in girls and 2.60% in boys (41). Of course, suicidal behavior is more prevalent in adolescents than in children (42). The association with age was also observed in Zhuhai City (China) (32). The rate of intentional injury in children and adolescents is increasing in several high-income countries (43, 44), stressing the need for proper suicide prevention measures.

A strength of the present study was the use of the NISS, a central system for injury registration, ensuring the completeness of the data. Still, the present study had limitations. It was performed at a single hospital covering a single district in China, limiting the generalizability of the results. Nevertheless, the study hospital is almost the first choice for injured patients in the study area. The analysis was retrospective, limiting the data to those available in the charts. Due to inevitable delays in data entry and compilation in large-scale databases and the impact of COVID-19, only data from the period of January 2016 to December 2019 were available for analysis. In the APC model analysis, the period and cohort factors were not statistically significant, probably due to the shorter duration of the observations. Although the injury outcome could be assessed, the exact morbidity was not compiled. The present study did not include rural/urban residency, which can influence the occurrence of animal bites and farm injuries.

In conclusion, our study underscores the persistent and substantial public health challenges posed by injuries among children and adolescents in the Xinglin District, China. The prevalence of injuries is primarily propelled by incidents of fall-related injuries and mechanical injuries, emphasizing the critical need for targeted preventive measures and interventions. Healthcare providers and policymakers can utilize this information to develop targeted injury prevention programs, enhance community awareness, and implement safety measures. By addressing the specific nature and causes of injuries identified in our study, interventions can be tailored to mitigate risks and reduce the overall burden of injuries among children and adolescents in the Xinglin District, with potential applicability to regions facing similar conditions.

## Data Availability

The original contributions presented in the study are included in the article/Supplementary Material, further inquiries can be directed to the corresponding author/s.
