# Supplementary material for: Epidemiology of injuries among children and adolescents from the Xinglin District in Xiamen, 2016–2019
Source: Front Pediatr. 2024 Sep 2;12:1387761. doi: 10.3389/fped.2024.1387761 (PMC11402704; doi:10.3389/fped.2024.1387761)
Supplement: Supplementary file 1 [file Datasheet1.pdf]

Table S1. Characteristics of the injuries among children and adolescents.

	0-4 years old	5-9 years old	10-14 years old	15-18 years old	P
	N=4834	N=3924	N=2671	N=1694	
**Intention**					<0.001
Unintentional accidents	4689 (97.00%)	3804 (96.94%)	2524 (94.50%)	1526 (90.08%)	
Adding insult to injury	103 (2.13%)	100 (2.55%)	130 (4.87%)	143 (8.44%)	
Other	42 (0.87%)	20 (0.51%)	17 (0.63%)	25 (1.47%)	
Uncertainty of intent	41 (0.85%)	18 (0.46%)	14 (0.52%)	9 (0.53%)	
Intentional self-harm	1 (0.02%)	2 (0.05%)	3 (0.11%)	16 (0.94%)	
**Body parts involved in the injury**					<0.001
Head	1988 (41.13%)	1079 (27.50%)	436 (16.32%)	305 (18.00%)	
Upper extremities	1539 (31.84%)	1275 (32.49%)	1077 (40.32%)	621 (36.66%)	
Lower extremities	559 (11.56%)	907 (23.11%)	777 (29.09%)	469 (27.69%)	
Trunk	157 (3.25%)	184 (4.69%)	148 (5.54%)	81 (4.78%)	

	0-4 years old	5-9 years old	10-14 years old	15-18 years old	P
Widespread injuries throughout the body/multiple sites	49 (1.01%)	61 (1.55%)	57 (2.13%)	83 (4.90%)	
Multiple sites	47 (0.97%)	58 (1.48%)	52 (1.95%)	74 (4.37%)	
Widespread injuries throughout the body	2 (0.04%)	3 (0.08%)	5 (0.19%)	9 (0.53%)	
Other	431 (8.92%)	393 (10.02%)	167 (6.25%)	120 (7.08%)	
Unknown	111 (2.30%)	25 (0.64%)	9 (0.34%)	15 (0.89%)	
**Body system(s) involved in injury**					<0.001
Respiratory system	261 (5.40%)	109 (2.78%)	88 (3.29%)	55 (3.25%)	
Digestive system	304 (6.29%)	171 (4.36%)	60 (2.25%)	37 (2.18%)	
Motor system	1903 (39.37%)	1950 (49.69%)	1725 (64.58%)	1027 (60.63%)	
Central nervous system	1087 (22.49%)	684 (17.43%)	276 (10.33%)	182 (10.74%)	
Other	1080 (22.34%)	900 (22.94%)	464 (17.37%)	312 (18.42%)	
Unknown	138 (2.85%)	67 (1.71%)	28 (1.05%)	25 (1.48%)	
Genitourinary system/multiple systems	61 (1.26%)	43 (1.10%)	30 (1.12%)	56 (3.31%)	

	0-4 years old	5-9 years old	10-14 years old	15-18 years old	P
Multiple systems	49 (1.01%)	31 (0.79%)	25 (0.94%)	50 (2.95%)	
Genitourinary system	12 (0.25%)	12 (0.31%)	5 (0.19%)	6 (0.35%)	
**Nature of injury**					<0.001
Contusions, abrasions	1767 (36.55%)	1337 (34.07%)	850 (31.82%)	526 (31.05%)	
Fracture	348 (7.20%)	494 (12.59%)	486 (18.20%)	187 (11.04%)	
Cerebral concussion, cerebral contusion, and laceration	237 (4.90%)	174 (4.43%)	61 (2.28%)	53 (3.13%)	
Internal organs injuries	48 (0.99%)	42 (1.07%)	28 (1.05%)	19 (1.12%)	
Sprains/Strains	682 (14.11%)	432 (11.01%)	428 (16.02%)	262 (15.47%)	
Other	33 (0.68%)	16 (0.41%)	13 (0.49%)	16 (0.94%)	
sharp instrument injury, bite injury, open injury	1518 (31.40%)	1373 (34.99%)	770 (28.83%)	581 (34.30%)	
Burns and scalds	71 (1.47%)	26 (0.66%)	13 (0.49%)	34 (2.01%)	
Unknown	130 (2.69%)	30 (0.76%)	22 (0.82%)	16 (0.94%)	
**Severity degree of injuries**					<0.001

	0-4 years old	5-9 years old	10-14 years old	15-18 years old	P
Mild	2966 (61.36%)	2018 (51.43%)	1233 (46.16%)	880 (51.95%)	
Moderate-severe	1868 (38.65%)	1906 (48.58%)	1438 (53.84%)	814 (48.06%)	
Moderate	1860 (38.48%)	1903 (48.50%)	1435 (53.73%)	810 (47.82%)	
Severe	8 (0.17%)	3 (0.08%)	3 (0.11%)	4 (0.24%)	
**Outcomes of injuries**					<0.001
Remain in hospital under observation	171(3.54%)	69(1.76%)	35(1.31%)	51(3.01%)	
Hospital discharge after treatment	4596 (95.08%)	3802 (96.89%)	2578 (96.52%)	1588 (93.74%)	
Hospitalization/transfer to another hospital/death/other	67 (1.38%)	53 (1.35%)	58 (2.17%)	55 (3.25%)	
Hospitalization	28 (0.58%)	36 (0.92%)	43 (1.61%)	54 (3.19%)	
Transfer to another hospital	34 (0.70%)	13 (0.33%)	14 (0.52%)	1 (0.06%)	
Death	2 (0.04%)	0	0	0	
Other	3 (0.06%)	4 (0.10%)	1 (0.04%)	0	

Categorical variables were expressed as n (%) and analyzed using the chi-squared test.

Table S2. Causes, locations, and categories of the injuries among children and adolescents.

	0-4 years old	5-9 years old	10-14 years old	15-18 years old	P
	n=4834	n=3924	n=2671	n=1694	
**Causes of injuries**					<0.001
Knife/sharp instrument injuries	234 (4.84%)	272 (6.93%)	182 (6.81%)	241 (14.23%)	
Fall down/fall	2667 (55.17%)	1866 (47.55%)	1346 (50.39%)	514 (30.34%)	
Animal injuries	420 (8.69%)	529 (13.48%)	335 (12.54%)	147 (8.68%)	
Blunt instrument injuries	836 (17.29%)	752 (19.16%)	469 (17.56%)	456 (26.92%)	
Non-motorized vehicle accident	106 (2.19%)	86 (2.19%)	69 (2.58%)	43 (2.54%)	
Motor vehicle accident	222 (4.59%)	258 (6.57%)	147 (5.50%)	156 (9.21%)	
Burns and scalds	71 (1.47%)	22 (0.56%)	12 (0.45%)	35 (2.07%)	
Unknown	167 (3.45%)	87 (2.22%)	81 (3.03%)	61 (3.60%)	
Other (combined)	111 (2.3%)	52 (1.33%)	30 (1.12%)	41 (2.42%)	
Poisoning	30 (0.62%)	5 (0.13%)	3 (0.11%)	11 (0.65%)	

	0-4 years old	5-9 years old	10-14 years old	15-18 years old	P
Drowning	2 (0.04%)	0	1 (0.04%)	1 (0.06%)	
Firearm injury	1 (0.02%)	1 (0.03%)	0	2 (0.12%)	
Sexual assault	2 (0.04%)	1 (0.03%)	0	0	
Apnea (suffocation)/suspension	1 (0.02%)	0	0	0	
**The major categories of unintentional injuries**					<0.001
Mechanical injuries	887 (18.35%)	881 (22.45%)	530 (19.84%)	617 (36.42%)	
Fall down/fall	2516 (52.05%)	1847 (47.07%)	1339 (50.13%)	516 (30.46%)	
Traffic injuries	318 (6.58%)	340 (8.66%)	212 (7.94%)	196 (11.57%)	
Animal injuries	426 (8.81%)	529 (13.48%)	333 (12.47%)	148 (8.74%)	
Human related injuries	233 (4.82%)	182 (4.64%)	165 (6.18%)	133 (7.85%)	
Apnea	114 (2.36%)	28 (0.71%)	23 (0.86%)	9 (0.53%)	
Poisoning	102 (2.11%)	17 (0.43%)	13 (0.49%)	32 (1.89%)	
Burns and scalds	72 (1.49%)	21 (0.54%)	13 (0.49%)	14 (0.83%)	

	0-4 years old	5-9 years old	10-14 years old	15-18 years old	P
Other (combined with unintentional injuries)	166 (3.43%)	79 (2.01%)	43 (1.61%)	29 (1.71%)	
Electric current, artificial visible light, ultraviolet light	1 (0.02%)	1 (0.03%)	0	3 (0.18%)	
Drowning	6 (0.12%)	6 (0.15%)	1 (0.04%)	2 (0.12%)	
Others	159 (3.29%)	72 (1.83%)	42 (1.57%)	24 (1.42%)	
**The subcategories of unintentional injuries**					<0.001
Traffic injuries	318 (6.58%)	340 (8.66%)	212 (7.94%)	196 (11.57%)	
Fall down/fall	2516 (52.05%)	1847 (47.07%)	1339 (50.13%)	516 (30.46%)	
Inanimate mechanical injuries	885 (18.31%)	877 (22.35%)	528 (19.77%)	617 (36.42%)	
Animal injuries (animals, excluding humans and plants, venomous animals)	409 (8.46%)	515 (13.12%)	315 (11.79%)	144 (8.50%)	
Animal injuries (human)	233 (4.82%)	182 (4.64%)	165 (6.18%)	133 (7.85%)	
Animal injuries (venomous animals)	17 (0.35%)	14 (0.36%)	18 (0.67%)	4 (0.24%)	
Apnea	114 (2.36%)	28 (0.71%)	23 (0.86%)	9 (0.53%)	
Burns and scalds	72 (1.49%)	21 (0.54%)	13 (0.49%)	14 (0.83%)	

	0-4 years old	5-9 years old	10-14 years old	15-18 years old	P
Poisoning (excluding animals)	102 (2.11%)	17 (0.43%)	13 (0.49%)	32 (1.89%)	
Other (combined with injuries)	166 (3.43%)	79 (2.01%)	43 (1.61%)	29 (1.72%)	
Drowning	6 (0.12%)	6 (0.15%)	1 (0.04%)	2 (0.12%)	
Electric current, artificial visible light, ultraviolet light	1 (0.02%)	1 (0.03%)	0	3 (0.18%)	
Others	159 (3.29%)	72 (1.83%)	42 (1.57%)	24 (1.42%)	
**Concomitant activities when injuries occurred**					<0.001
Leisure activities	2699 (55.83%)	1819 (46.36%)	763 (28.57%)	389 (22.96%)	
Unspecified activities	984 (20.36%)	869 (22.15%)	601 (22.50%)	359 (21.19%)	
Other types of work	609 (12.60%)	585 (14.91%)	510 (19.09%)	223 (13.16%)	
Life activities	473 (9.78%)	287 (7.31%)	152 (5.69%)	89 (5.25%)	
Other (combined with activities when injuries occurred)	69 (1.43%)	364 (9.28%)	645 (24.15%)	634 (37.43%)	
Physical activities	69 (1.43%)	364 (9.28%)	645 (24.15%)	308 (18.18%)	
Work	0	0	0	326 (19.24%)	

	0-4 years old	5-9 years old	10-14 years old	15-18 years old	P
**Injuries related to product quality**					<0.001
Used as usual but had a sudden accident	1978 (40.92%)	1683 (42.89%)	1245 (46.61%)	764 (45.10%)	
Not sure	500 (10.34%)	416 (10.60%)	259 (9.70%)	148 (8.74%)	
Not filled	1612 (33.35%)	1162 (29.61%)	677 (25.35%)	514 (30.34%)	
Other (combined with other conditions)	744 (15.39%)	663 (16.90%)	490 (18.34%)	268 (15.82%)	
Other	465 (9.62%)	539 (13.74%)	420 (15.72%)	206 (12.16%)	
Improper use	275 (5.69%)	121 (3.08%)	70 (2.62%)	62 (3.66%)	
Related to product quality	4 (0.08%)	3 (0.08%)	0	0	
**The location where the injury occurred**					<0.001
At home	3352 (69.34%)	1439 (36.67%)	546 (20.44%)	271 (16.00%)	
On the streets and highways	500 (10.34%)	549 (13.99%)	400 (14.98%)	298 (17.59%)	
At the resident public service facilities	433 (8.96%)	555 (14.14%)	253 (9.47%)	115 (6.79%)	
At schools, other institutions, and public administration regions	272 (5.63%)	959 (24.44%)	912 (34.14%)	347 (20.48%)	

	0-4 years old	5-9 years old	10-14 years old	15-18 years old	P
At the sports and exercise regions	98 (2.03%)	280 (7.14%)	471 (17.63%)	242 (14.29%)	
At the trade and service regions	93 (1.92%)	70 (1.78%)	40 (1.50%)	105 (6.20%)	
At unspecified places	59 (1.22%)	27 (0.69%)	15 (0.56%)	12 (0.71%)	
Other (combined the location where the injury occurred)	27 (0.55%)	45 (1.15%)	34 (1.27%)	304 (17.95%)	
In the industry and construction regions	20 (0.41%)	20 (0.51%)	16 (0.60%)	298 (17.59%)	
At the farms	1 (0.02%)	13 (0.33%)	6 (0.22%)	4 (0.24%)	
At other specially designated regions	6 (0.12%)	12 (0.31%)	12 (0.45%)	2 (0.12%)	

Categorical variables were expressed as n (%) and analyzed using the chi-squared test.

Table S3. Characteristics of patients with fall down/fall injuries.

	0-4 years old	5-9 years old	10-14 years old	15-18 years old	P
	n=2516	n=1847	n=1339	n=516	
**Gender**					<0.001
Male	1593 (63.31%)	1259 (68.16%)	1028 (76.77%)	384 (74.42%)	
Female	923 (36.69%)	588 (31.84%)	311 (23.23%)	132 (25.58%)	
**Causes of injuries**					
Unintentional accidents	2516 (100.00%)	1847 (100.00%)	1339 (100.00%)	515 (99.81%)	-
**The location where the injury occurred**					<0.001
At home	1840 (73.13%)	631 (34.16%)	150 (11.20%)	48 (9.30%)	
At the resident public service facilities	230 (9.14%)	284 (15.38%)	135 (10.08%)	32 (6.20%)	
At schools, other institutions, and public administration regions	176 (7.00%)	505 (27.34%)	524 (39.13%)	146 (28.29%)	
At the sports and exercise regions	75 (2.98%)	216 (11.69%)	358 (26.74%)	170 (32.95%)	
On the streets and highways	132 (5.25%)	154 (8.34%)	137 (10.23%)	77 (14.92%)	

	0-4 years old	5-9 years old	10-14 years old	15-18 years old	P
At the trade and service regions	53 (2.11%)	38 (2.06%)	22 (1.64%)	16 (3.10%)	
Other (combined the location where the injury occurred)	10 (0.40%)	19 (1.03%)	13 (0.97%)	27 (5.23%)	
In the industry and construction regions	4 (0.16%)	8 (0.43%)	6 (0.45%)	27 (5.23%)	
At the farms	0	3 (0.16%)	1 (0.07%)	0	
At other specially designated regions	1 (0.04%)	5 (0.27%)	5 (0.37%)	0	
At unspecified places	5 (0.20%)	3 (0.16%)	1 (0.07%)	0	
**Concomitant activities when injuries occurred**					<0.001
Leisure activities	1462 (58.11%)	817 (44.23%)	316 (23.60%)	101 (19.57%)	
Unspecified activities	419 (16.65%)	350 (18.95%)	244 (18.22%)	93 (18.02%)	
Life activities	237 (9.42%)	115 (6.23%)	52 (3.88%)	20 (3.88%)	
Physical activities	57 (2.27%)	278 (15.05%)	505 (37.71%)	214 (41.47%)	
Other (combined with activities when injuries occurred)	341 (13.55%)	287 (15.54%)	222 (16.58%)	88 (17.05%)	
Other types of work	341 (13.55%)	287 (15.54%)	222 (16.58%)	59 (11.43%)	

	0-4 years old	5-9 years old	10-14 years old	15-18 years old	P
Work	0	0	0	29 (5.62%)	
**Severity degree of injuries**					<0.001
Mild	1412 (56.12%)	779 (42.18%)	491 (36.67%)	221 (42.83%)	
Moderate-severe	1104 (43.88%)	1068 (57.82%)	848 (63.33%)	295 (57.17%)	
Moderate	1103 (43.84%)	1066 (57.72%)	847 (63.26%)	293 (56.78%)	
Severe	1 (0.04%)	2 (0.11%)	1 (0.07%)	2 (0.39%)	
**Body parts involved in the injury**					<0.001
Head	1360 (54.05%)	617 (33.41%)	172 (12.85%)	74 (14.34%)	
Upper extremities	705 (28.02%)	664 (35.95%)	640 (47.80%)	152 (29.46%)	
Lower extremities	160 (6.36%)	371 (20.09%)	417 (31.14%)	239 (46.32%)	
Trunk	57 (2.27%)	56 (3.03%)	52 (3.88%)	24 (4.65%)	
Other(combined)	234 (9.30%)	139 (7.53%)	58 (4.33%)	27 (5.23%)	
Multiple sites	9 (0.36%)	18 (0.97%)	21 (1.57%)	13 (2.52%)	

	0-4 years old	5-9 years old	10-14 years old	15-18 years old	P
Widespread injuries throughout the body	0	1 (0.05%)	1 (0.07%)	4 (0.78%)	
Other	221 (8.78%)	120 (6.50%)	36 (2.69%)	9 (1.74%)	
Unknown	4 (0.16%)	0	0	1 (0.19%)	
**Body system(s) involved in injury**					<0.001
Motor system	904 (35.93%)	1055 (57.12%)	1060 (79.16%)	402 (77.91%)	
Central nervous system	794 (31.56%)	429 (23.23%)	124 (9.26%)	47 (9.11%)	
Digestive system	208 (8.27%)	85 (4.60%)	29 (2.17%)	8 (1.55%)	
Respiratory system	43 (1.71%)	15 (0.81%)	23 (1.72%)	9 (1.74%)	
Other	517 (20.55%)	226 (12.24%)	86 (6.42%)	35 (6.78%)	
Unknown	45 (1.79%)	20 (1.08%)	10 (0.75%)	7 (1.36%)	
Genitourinary system/multiple systems	5 (0.20%)	17 (0.92%)	7 (0.52%)	8 (1.55%)	
Genitourinary system	1 (0.04%)	4 (0.22%)	1 (0.07%)	1 (0.19%)	
Multiple systems	4 (0.16%)	13 (0.70%)	6 (0.45%)	7 (1.36%)	

	0-4 years old	5-9 years old	10-14 years old	15-18 years old	P
**Nature of injury**					<0.001
Contusions, abrasions	926 (36.80%)	565 (30.59%)	366 (27.33%)	136 (26.36%)	
Sharp instrument injury, Bite injury, Open injury	712 (28.30%)	402 (21.77%)	171 (12.77%)	84 (16.28%)	
Sprains/Strains	383 (15.22%)	322 (17.43%)	329 (24.57%)	185 (35.85%)	
Fracture	314 (12.48%)	434 (23.50%)	428 (31.96%)	98 (18.99%)	
Other (combined with injuries)	181 (7.19%)	124 (6.71%)	45 (3.36%)	13 (2.52%)	
Cerebral concussion, cerebral contusion and laceration	167 (6.64%)	105 (5.68%)	29 (2.17%)	11 (2.13%)	
Burns and Scalds	3 (0.12%)	2 (0.11%)	1 (0.07%)	0	
Internal organs injuries	2 (0.08%)	12 (0.65%)	9 (0.67%)	1 (0.19%)	
Other	5 (0.20%)	4 (0.22%)	2 (0.15%)	0	
Unknown	4 (0.16%)	1 (0.05%)	4 (0.30%)	1 (0.19%)	
**Causes of Injuries**					0.030
Fall down/fall	2495 (99.17%)	1821 (98.59%)	1323 (98.81%)	504 (97.67%)	

	0-4 years old	5-9 years old	10-14 years old	15-18 years old	P
Other (combined with injuries)	21 (0.83%)	26 (1.41%)	16 (1.19%)	12 (2.33%)	
Blunt instrument injuries	17 (0.68%)	16 (0.87%)	12 (0.90%)	8 (1.55%)	
Non-motorized vehicle accident	1 (0.04%)	2 (0.11%)	1 (0.07%)	0	
Motor vehicle accident	0	0	1 (0.07%)	2 (0.39%)	
Knife/sharp instrument injuries	1 (0.04%)	3 (0.16%)	0	1 (0.19%)	
Unknown	2 (0.08%)	5 (0.27%)	2 (0.15%)	1 (0.19%)	

Categorical variables were expressed as n (%) and analyzed using the chi-squared test.

Table S4. Characteristics of patients with mechanical injuries.

	0-4 years old	5-9 years old	10-14 years old	15-18 years old	P
	n=887	n=881	n=530	n=617	
**Gender**					<0.001
Male	589 (66.40%)	638 (72.42%)	414 (78.11%)	498 (80.71%)	
Female	298 (33.60%)	243 (27.58%)	116 (21.89%)	119 (19.29%)	
**Causes of injuries**					<0.001
Unintentional accidents	879 (99.10%)	857 (97.28%)	505 (95.28%)	565 (91.57%)	
Intentional accidents	8 (0.90%)	24 (2.72%)	25 (4.72%)	52 (8.43%)	
Intentional self-harm	1 (0.11%)	1 (0.11%)	0	4 (0.65%)	
Adding insult to injury	5 (0.56%)	22 (2.50%)	24 (4.53%)	47 (7.62%)	
Uncertainty of intent	2 (0.23%)	1 (0.11%)	1 (0.19%)	1 (0.16%)	
**Types of injuries**					-
Inanimate mechanical injuries	885 (99.77%)	877 (99.55%)	528 (99.62%)	617 (100.00%)	

	0-4 years old	5-9 years old	10-14 years old	15-18 years old	P
Animals/Plants injuries	2 (0.23%)	4 (0.45%)	2 (0.38%)	0	
**The location where the injury occurred**					<0.001
At home	622 (70.12%)	344 (39.05%)	107 (20.19%)	80 (12.97%)	
At the resident public service facilities	77 (8.68%)	122 (13.85%)	51 (9.62%)	31 (5.02%)	
At schools, other institutions, and public administration regions	53 (5.98%)	274 (31.10%)	229 (43.21%)	119 (19.29%)	
At the sports and exercise regions	19 (2.14%)	54 (6.13%)	87 (16.42%)	54 (8.75%)	
On the streets and highways	58 (6.54%)	44 (4.99%)	28 (5.28%)	21 (3.40%)	
Other (combined the location where the injury occurred)	58 (6.54%)	43 (4.88%)	28 (5.28%)	312 (50.57%)	
At the trade and service regions	29 (3.27%)	18 (2.04%)	7 (1.32%)	62 (10.05%)	
In the industry and construction regions	11 (1.24%)	8 (0.91%)	7 (1.32%)	243 (39.38%)	
At the farms	0	5 (0.57%)	3 (0.57%)	2 (0.32%)	
At other specially designated regions	4 (0.45%)	6 (0.68%)	7 (1.32%)	2 (0.32%)	
At unspecified places	14 (1.58%)	6 (0.68%)	4 (0.75%)	3 (0.49%)	

	0-4 years old	5-9 years old	10-14 years old	15-18 years old	P
**Concomitant activities when injuries occurred**					<0.001
Physical activities	10 (1.13%)	65 (7.38%)	103 (19.43%)	69 (11.18%)	
Leisure activities	519 (58.51%)	494 (56.07%)	193 (36.42%)	126 (20.42%)	
Life activities*	97 (10.94%)	65 (7.38%)	32 (6.04%)	26 (4.21%)	
Unspecified activities	176 (19.84%)	131 (14.87%)	71 (13.40%)	60 (9.72%)	
Work/Others	85 (9.58%)	126 (14.30%)	131 (24.72%)	336 (54.30%)	
Work	0	0	0	255 (41.33%)	
Other types of work	85 (9.58%)	126 (14.30%)	131 (24.72%)	80 (12.97%)	
Others	0	0	0	1 (0.16%)	
**Severity degree of injuries**					0.002
Mild	567 (63.92%)	549 (62.32%)	300 (56.60%)	344 (55.75%)	
Moderate-severe	320 (36.07%)	332 (37.68%)	230 (43.40%)	273 (44.25%)	
Moderate	319 (35.96%)	332 (37.68%)	230 (43.40%)	273 (44.25%)	

	0-4 years old	5-9 years old	10-14 years old	15-18 years old	P
Severe	1 (0.11%)	0	0	0	
**Body parts involved in the injury**					<0.001
Head	208 (23.45%)	238 (27.01%)	115 (21.70%)	92 (14.91%)	
Upper extremities	349 (39.35%)	241 (27.36%)	200 (37.74%)	313 (50.73%)	
Lower extremities	143 (16.12%)	177 (20.09%)	108 (20.38%)	108 (17.50%)	
Trunk	37 (4.17%)	41 (4.65%)	34 (6.42%)	24 (3.89%)	
Other	126 (14.21%)	178 (20.20%)	71 (13.40%)	72 (11.67%)	
Widespread injuries throughout the body or multiple sites injuries/unknown	24 (2.71%)	6 (0.68%)	2 (0.38%)	8 (1.30%)	
Widespread injuries throughout the body	0	1 (0.11%)	0	0	
Multiple sites	4 (0.45%)	1 (0.11%)	1 (0.19%)	8 (1.30%)	
Unknown	20 (2.25%)	4 (0.45%)	1 (0.19%)	0	
**Body system(s) involved in injury**					<0.001
Motor system	400 (45.10%)	331 (37.57%)	264 (49.81%)	373 (60.45%)	

	0-4 years old	5-9 years old	10-14 years old	15-18 years old	P
Central nervous system	122 (13.75%)	134 (15.21%)	72 (13.58%)	46 (7.46%)	
Digestive system	53 (5.98%)	23 (2.61%)	8 (1.51%)	8 (1.30%)	
Other	279 (31.45%)	353 (40.07%)	158 (29.81%)	155 (25.12%)	
Respiratory system/genitourinary system/multiple systems or unknown	33 (3.72%)	40 (4.54%)	28 (5.28%)	35 (5.67%)	
Respiratory system	5 (0.56%)	22 (2.50%)	20 (3.77%)	18 (2.92%)	
Genitourinary system	5 (0.56%)	2 (0.23%)	1 (0.19%)	1 (0.16%)	
Multiple systems	3 (0.34%)	0	3 (0.57%)	6 (0.97%)	
Unknown	20 (2.25%)	16 (1.82%)	4 (0.75%)	10 (1.62%)	
**Nature of injury**					<0.001
Sharp instrument injury, bite injury, open injury	363 (40.92%)	400 (45.40%)	220 (41.51%)	306 (49.59%)	
Contusions, abrasions	361 (40.70%)	371 (42.11%)	216 (40.75%)	192 (31.12%)	
Sprains/strains	78 (8.79%)	40 (4.54%)	44 (8.30%)	34 (5.51%)	
Fracture	18 (2.03%)	27 (3.06%)	28 (5.28%)	54 (8.75%)	

	0-4 years old	5-9 years old	10-14 years old	15-18 years old	P
Cerebral concussion, cerebral contusion, and laceration	18 (2.03%)	27 (3.06%)	13 (2.45%)	10 (1.62%)	
Other (combined with injuries)					
Burns and scalds	0	3 (0.34%)	1 (0.19%)	18 (2.92%)	
Internal organs injuries	18 (2.03%)	6 (0.68%)	2 (0.38%)	0	
Other	6 (0.68%)	4 (0.45%)	2 (0.38%)	1 (0.16%)	
Unknown	25 (2.82%)	3 (0.34%)	4 (0.75%)	2 (0.32%)	
**Causes of injuries**					<0.001
Blunt instrument injuries	551 (62.12%)	560 (63.56%)	312 (58.87%)	343 (55.59%)	
Knife/sharp instrument injuries	219 (24.69%)	266 (30.19%)	177 (33.40%)	236 (38.25%)	
Unknown	42 (4.74%)	19 (2.16%)	27 (5.09%)	13 (2.11%)	
Other (combined with injuries)	75 (8.46%)	36 (4.09%)	14 (2.64%)	25 (4.05%)	
Non-motorized vehicle accident	4 (0.45%)	1 (0.11%)	0	0	
Firearm injury	0	0	0	1 (0.16%)	

	0-4 years old	5-9 years old	10-14 years old	15-18 years old	P
Motor vehicle accident	2 (0.23%)	3 (0.34%)	2 (0.38%)	1 (0.16%)	
Fall down/fall	50 (5.64%)	19 (2.16%)	5 (0.94%)	2 (0.32%)	
Burns and scalds	2 (0.23%)	0	1 (0.19%)	18 (2.92%)	
Other	17 (1.92%)	13 (1.48%)	6 (1.13%)	3 (0.49%)	

Categorical variables were expressed as n (%) and analyzed using the chi-squared test.

Supplementary Material 1

ICD10 injury class codes

V01-X59 Accidents, V01-V99 Traffic accidents, V01-V09 Pedestrians injured in traffic accidents, V10-V19 Cyclists injured in traffic accidents, V20-V29 Motorcyclists injured in traffic accidents, V30-V39 Occupants of three-wheeled motor vehicles injured in traffic accidents, V40-V49 Occupants of automobiles injured in traffic accidents, V50- V59 Passengers of pickup trucks or vans injured in transportation accidents, V60-V69 Occupants of heavy transportation vehicles injured in transportation accidents, V70-V79 Bus passengers injured in transportation accidents, V80-V89 Other land traffic accidents, V90-V94 Water transportation accidents, V95-V97 Aerospace transportation accidents, V98-V99 Other and unspecified transportation accidents W00-X59 Other external causes of injury, W00-W19 Fall down/fall, W20-W49 Exposure to inanimate mechanical forces, W50-W64 Exposure to dynamic mechanical forces, W65-W74 Unintentional drowning and diving, W75-W84 Other unintentional respiratory threats, W85-W99 Exposure to electrical currents, radiation, and extremes of ambient air temperatures and pressures, X00- X09 Exposure to smoke, fire and flame, X10-X19 Exposure to heat and hot substances, X20-X29 Exposure to poisonous plants and animals, X30-X39 Exposure to the forces of nature, X40-X49 Unintentional poisoning due to exposure to poisonous substances, X50-X57 Overexertion, travel, and poverty, X58-X59 Unintentional exposure to other unspecified factors, X60-X84 Intentional self-harm, X85-Y09 Assault, Y10-Y34 Events of Unspecified Intent, Y35-Y36 Legal Interventions and Actions of War, Y85-Y89 External Cause Sequelae of Morbidity and Mortality, and Y90-Y98 Associated with Other Locally Categorized Causes of Morbidity and Mortality; Outcomes of Injury Supplementary Factors are

coded as S00 - S09 Head Injury, S10 - S19 Neck Injuries, S20 - S29 Chest Injuries, S30 - S39 Abdominal, Lower Back, Lumbar Spine, and Pelvic Injuries, S40 - S49 Shoulder and Upper Arm Injuries, S50 - S59 Elbow and Forearm Injuries, S60 - S69 Wrist and Hand Injuries, S70 - S79 Hip and Thigh Injuries, S80 - S89 Knee and Lower Leg Injuries, and S90 - S99 Ankle and Foot Injuries, T00-T07 Multiple site injuries, T08-T14 Unspecified injuries to trunk, extremities, or body parts, T15-T19 Effects of foreign matter entering through natural orifices, T20-T32 Burns and corrosion, T20-T25 Burns and corrosion of external body surfaces according to site specifications, T26-T28 Burns and corrosion confined to the eyes and internal organs, T29-T32 Burns and corrosion of multiple unspecified body parts,T33-T35 Frostbite, T36-T50 Poisoning by drugs, medications, and biological substances, T51-T65 Toxic effects primarily of nonmedicinal substances, T66-T78 Other unspecified external causative effects, T70-T79 Certain early complications of trauma, T90-T98 Sequelae of traumatic injuries, poisonings, and other external causative consequences.

ICD10 class codes of iatrogenic injury

Y40-Y84 Complications of Medical-Surgical Nursing, T80-T88 Outcome Codes, T98.3 Complications of Surgery and Medical Care.

Supplementary Material 2

Clinical classifications of the injuries:

Major categories of injuries		Subcategories of injuries	
injuries category	Code	Injuries category	Code
mechanical Injuries	001	inanimate mechanical injuries	03
		animal injuries (plants)	06
fall down/fall	002	fall down/fall	02
traffic injuries	003	traffic injuries	01
animal injuries	004	animal injuries (animals, excluding humans and plants and venomous animals)	04
		animal injuries (venomous animals)	05
human-related injuries	005	animal injuries (human)	07
apnea	006	apnea	09

poisoning	007	poisoning (excluding animals)	11
burns and scalds	008	burns and scalds	10
electric current, artificial visible light, and ultraviolet light	009	electric current, artificial visible light, and ultraviolet light	12
drowning	010	drowning	08
heat stroke, frostbite, and lightning strikes (natural forces)	011	heat stroke, frostbite, and lightning strikes (natural forces)	13
medical-related injuries	012	medical-related injuries	14
others	013	others	15
